# Fabrication and performance of highly stacked GeSi nanowire field effect transistors

**DOI:** 10.1038/s44172-023-00126-8

**Published:** 2023-11-01

**Authors:** Yu-Rui Chen, Yi-Chun Liu, Hsin-Cheng Lin, Chien-Te Tu, Tao Chou, Bo-Wei Huang, Wan-Hsuan Hsieh, Shee-Jier Chueh, C. W. Liu

**Affiliations:** 1 Graduate Institute of Electronics Engineering, National Taiwan University, Taipei, 106 Taiwan; 2 Graduate School of Advanced Technology, National Taiwan University, Taipei, 106 Taiwan; 3 Graduate Institute of Photonics and Optoelectronics, National Taiwan University, Taipei, 106 Taiwan

**Keywords:** Electrical and electronic engineering, Nanowires, Electronic devices

## Abstract

Horizontal gate-all-around field effect transistors (GAAFETs) are used to replace FinFETs due to their good electrostatics and short channel control. Highly stacked nanowire channels are widely believed to enhance drive current of these devices and improve overall transistor density due to their small footprint. Here we demonstrate the fabrication and characterization of nanowire FETs with stacked 16 Ge_0.95_Si_0.05_ nanowires and stacked 12 Ge_0.95_Si_0.05_ nanowires without parasitic channels. The device has the high on current (I_ON_) of 190 μA per stack (9400 μA/μm per channel footprint) at overdrive voltage (V_OV_) = drain-source voltage (V_DS_) = 0.5 V and the high maximum transconductance (G_m,max_) of 490μS (24000μS/μm) at V_DS_ = 0.5 V among reported Si/Ge/GeSi 3D nFETs. Note that the transistor performance can be evaluated by the delay, which is depicted as CV/I. If the transistor I_ON_ is improved, the delay of standard cell can be reduced, leading to faster operation of the circuit. The subthreshold slope reduction and I_ON_/I_OFF_ improvement are achieved by the parasitic channel removal. In technology computer aided design (TCAD) simulation, the wrap around contacts are useful to reduce the current difference between the channels. With the proper design of transistor height, the gate delay can be also improved.

## Abstract (web summary)

Gate-all-round field effect transistors (FETs) with channels fabricated from highly stacked nanowires can enhance the drive current of such devices for a fixed footprint. Chen, Liu, and colleagues fabricated FETs with as many as 16 highly stacked Ge_0.95_Si_0.05_ nanowires and 12 nanowires without parasitic channels using wet etching.

## Introduction

The gate-all-around (GAA) devices are used to replace FinFETs for the advanced technology nodes thanks to the superior electrostatics and short channel control^1–6^. The GAA structure with channel stacking can further enhance the drive current for a fixed footprint to achieve high performance and area scaling^4^. To improve the I_ON_, most efforts are focused on high mobility channels such as the recently commercialized 5 nm node^5,6^. Ge is an attractive option for the high mobility channel to boost the drive current thanks to its intrinsic higher mobility than Si^7^. Alternatively, the highly stacked channels to further increase I_ON_ is also a knob to achieve the improvement. The systematic work to increase number of vertically stacked channels for I_ON_ enhancement with decreasing gate delay is presented in this work. The high etching selectivity between channels and sacrificial layers (SLs) are required for highly stacked channels. Recently, the radical-based highly selective isotropic dry etching was reported to form the highly stacked channels^8–10^. A simple isotropic wet etching by H_2_O_2_ has been reported to form stacked 2 nanowires without high energy ion damage in our previous work^11,12^. Moreover, the stacked 7 Ge_0.95_Si_0.05_ nanowires with high performance by wet etching have been reported^13,14^. To further improve the I_OFF_, NH_4_OH + H_2_O_2_ wet etching was used to remove parasitic channels^14^.

In this work, the highest stacked 16 Ge_0.95_Si_0.05_ nanowires and stacked 12 Ge_0.95_Si_0.05_ nanowires without parasitic channels are demonstrated by the low temperature epitaxy and wet etching. The isotropic wet etching can reach the sufficient selectivity to fabricate the highly stacked Ge_0.95_Si_0.05_ nanowires using n^+^Ge SLs. Note that Si content as low as 5% can reach the etching selectivity between channels and sacrificial layers. Due to the excessive etching in parasitic channel removal, only the stacked 12 nanowires are remained. As compared with our previous work^13^, higher I_ON_ per stack (per footprint), larger G_m,max_ per stack (per footprint), SS reduction, and I_ON_/I_OFF_ improvement are achieved by increasing number of stacked channels and parasitic channel removal.

## Results

### Device structure and epilayer

**Fig. 1:**
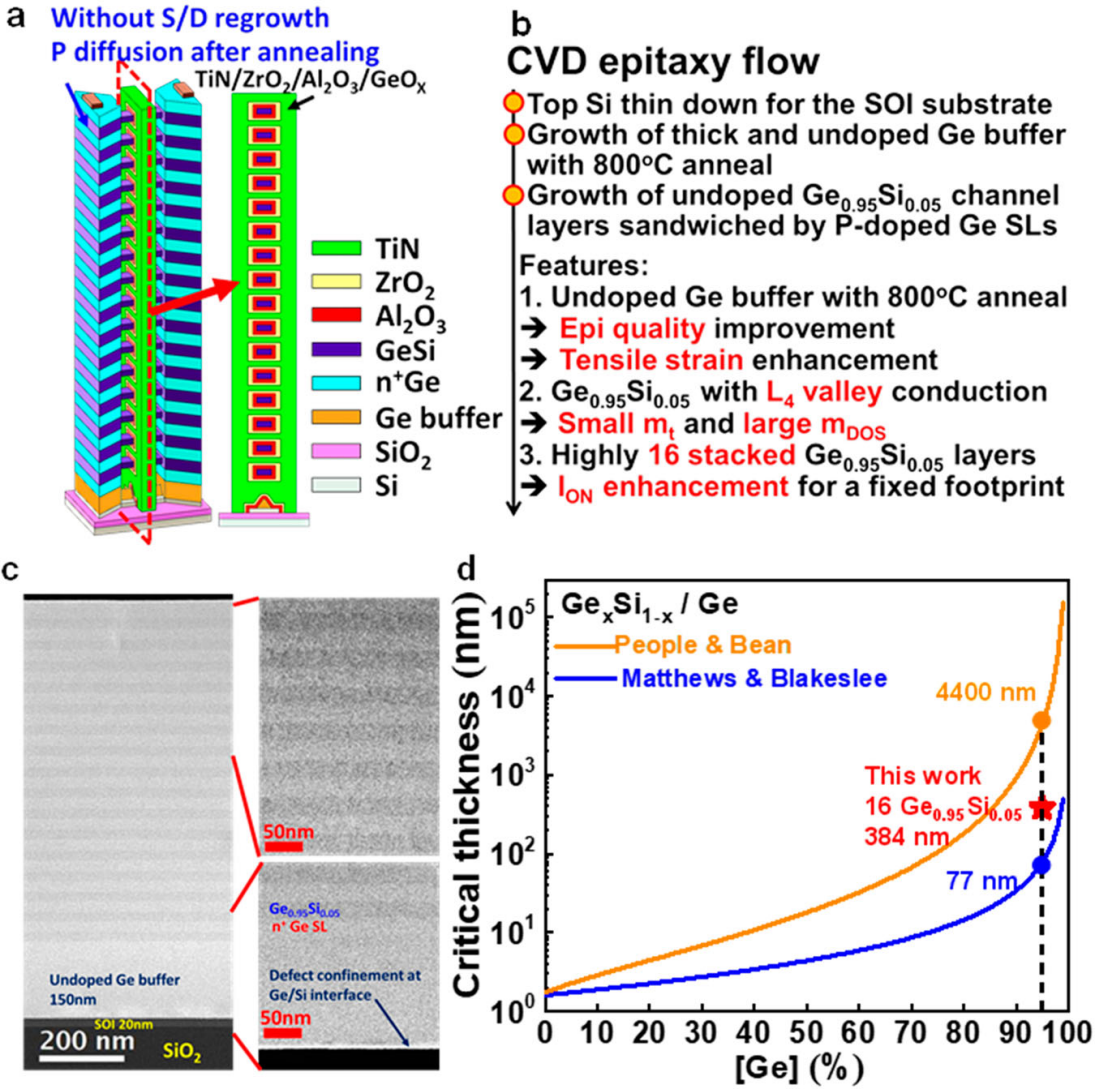
Device structure and epilayer fabrication. **a** 3D schematics of the stacked 16 Ge_0.95_Si_0.05_ nanowires. **b** CVD epitaxy flow with the highlighted features (red). **c** TEM-HAADF of the epilayers. 16 undoped Ge_0.95_Si_0.05_ layers are sandwiched by 17 P-doped Ge sacrificial layers (SLs). Defects are confined at the Ge/Si interface. Note that the 25 nm heavily P-doped Ge SL and the 24 nm undoped Ge_0.95_Si_0.05_ channel layer were grown on the Ge buffer 16 times repeatedly, followed by the top 44 nm heavily P-doped Ge SL deposition. **d** Critical thickness versus Ge content. Note that the material parameter of GeSi is linear combination of Ge and Si (virtual crystal approximation). The critical thickness by Matthews and Blakeslee theory (thermal equilibrium, blue line) of Ge_0.95_Si_0.05_ deposited on Ge is 77 nm (blue circle). The critical thickness by People and Bean theory (metastable, orange line) of Ge_0.95_Si_0.05_ deposited on Ge is 4400 nm (orange circle). Our growth temperature of CVD Ge_0.95_Si_0.05_ and n^+^Ge SL are maintained at 350 °C for metastable states. The total thickness of stacked 16 undoped Ge_0.95_Si_0.05_ channels is 384 nm (red star), lower than critical thickness at metastable state.

The 3D schematic of the stacked 16 Ge_0.95_Si_0.05_ nanowire nFETs are shown in Fig. 1a. The current flows along (110) direction. The highest stacked channels without source/drain (S/D) regrowth is demonstrated. The doping of S/D is obtained by the heavily P-doped Ge sacrificial layers (SLs), which are annealed during the device fabrication to have P diffusion. The process flow of chemical vapor deposition (CVD) epitaxy is shown in Fig. 1b. The top Si of a 200 mm silicon-on-insulator (SOI) substrate was thinned down from 70 to 20 nm by the oxidation in a vertical furnace and dipped into the buffered oxide etchant. After HF dipping to remove the native oxide, a 200 mm SOI substrate was loaded into a rapid thermal chemical vapor deposition system with a cold-wall quartz chamber, followed by 1100 °C H_2_ baking at 80 torr to further remove the residual native oxide on the SOI surface. GeH_4_, SiH_4_, and PH_3_ were used as the precursors for the following epitaxial growth process. The 150 nm undoped Ge buffer was grown on an SOI wafer at 375 °C using the GeH_4_ precursor in H_2_ ambient at 40 torr. Additional in-situ annealing at 800 °C for 3 min in H_2_ ambient after the Ge buffer growth was used to confine the dislocations near the Ge buffer/SOI interface and to improve the quality of the Ge buffer. For Ge_0.95_Si_0.05_ channels, the 25 nm heavily P-doped Ge SL and the 24 nm undoped Ge_0.95_Si_0.05_ channel layer were grown on the Ge buffer 16 times repeatedly, followed by the top 44 nm heavily P-doped Ge SL deposition. The thick top n^+^Ge SL is designed to protect the disappearance of a top channel from etching away after channel release. The n^+^Ge SLs were grown using GeH_4_ and PH_3_ precursors and the Ge_0.95_Si_0.05_ channel layers were grown using GeH_4_ and SiH_4_ precursors both at 350 °C in H_2_ ambient at 100 torr. There are a total of 34 epilayers (the undoped Ge buffer + 16 undoped GeSi channel layers + 17 heavily P-doped Ge SLs). The undoped Ge_0.95_Si_0.05_ channel layers can suppress the impurity scattering for the high electron mobility, and the heavily P-doped Ge SLs can reduce the S/D resistance. The transmission electron microscopy high angle annular dark field (TEM-HAADF) image of the as-grown epilayers are shown in Fig. 1c. The low-temperature epitaxial growth of channel layers and SLs ensures the entire epilayers metastable without dislocations in the channels and precisely controls the epilayers with good thickness and concentration uniformities both vertically and horizontally. Fig. 1d shows how we achieve high quality epilayers without dislocations in the channels. The total thickness of channel layers should be less than critical thickness for high quality epilayers. The critical thickness versus Ge content is shown is Fig. 1d. The critical thickness of Matthews and Blakeslee theory (thermal equilibrium, high temperature growth) for Ge_0.95_Si_0.05_ deposited on Ge is 77 nm, while People and Bean theory (metastable, low temperature growth) has a critical thickness of 4400 nm. In this work, the growth temperature of CVD Ge_0.95_Si_0.05_ and n^+^Ge SL is maintained at 350 °C for the metastable state. The total thickness for stacked 16 undoped Ge_0.95_Si_0.05_ channels is 384 nm, which is lower than metastable critical thickness. The low-temperature epitaxial growth of channel layers and SLs ensures the entire epilayers metastable without dislocations.

### Material analysis of epilayers

**Fig. 2:**
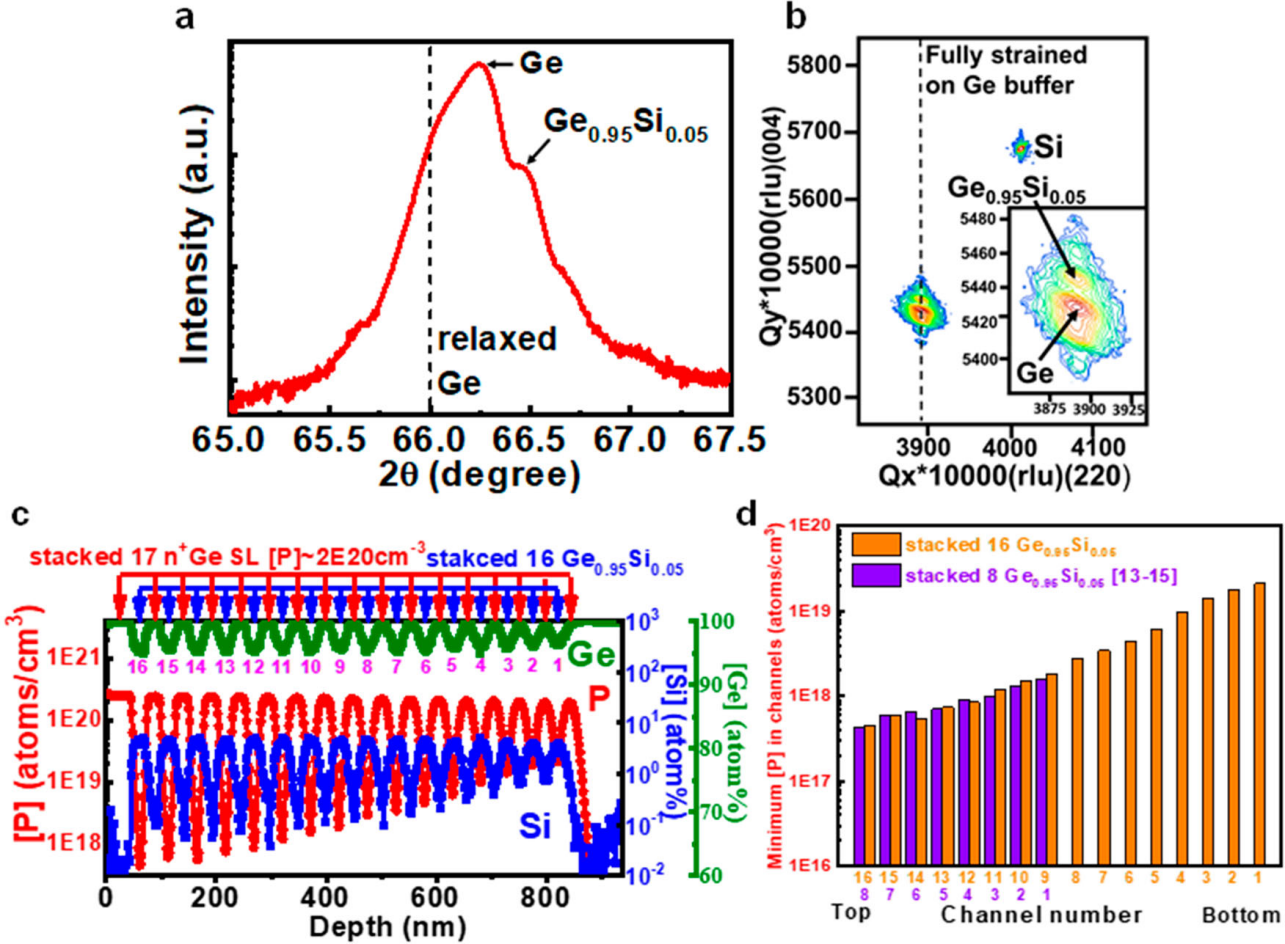
Material analysis of CVD epitaxy. High-resolution X-ray diffraction (**a**), reciprocal space mapping (**b**), and secondary ion mass spectrometry (**c**) of the epilayers. **d** Minimum [P] vs channel number. The Ge and Ge_0.95_Si_0.05_ are 0.2% and 0.4% tensily strained, respectively. Note that tensile strain in Ge is caused by the mismatch of thermal expansion coefficients between Ge and Si. The [P] in Ge SLs is as high as ~2 × 10^20^ cm^−3^ for low S/D resistance, and the minimum [P] in Ge_0.95_Si_0.05_ channels are from ~4 × 10^17^ cm^−3^ to ~2 × 10^19^ cm^−3^. Note that the Ge, P, and Si are in green, red and blue lines. The minimum [P] increases from top to bottom due to P diffusion, consistent with the diffusion time in the reaction chamber^13–15^. Note that the orange and purple bars correspond to stacked 16 Ge_0.95_Si_0.05_ and stacked 8 Ge_0.95_Si_0.05_, respectively.

The as-grown epilayers with stacked 16 Ge_0.95_Si_0.05_ channels were analyzed by the high-resolution X-ray diffraction with ω − 2θ scan of (004) reflections (Fig. 2a). The shifting peak to higher 2θ as compared to relaxed Ge indicates the tensile strain in epitaxial Ge buffer. The Ge buffer is 0.2% tensily strained on Si. Note that the tensile strain in Ge buffer is caused by the mismatch of thermal expansion coefficients between Ge and Si. The shoulder in high-resolution X-ray diffraction is the diffraction by Ge_0.95_Si_0.05_. To further analyze the strain of epitaxial layers, the reciprocal space mapping was used. The Ge_0.95_Si_0.05_ channels are fully tensily strained on the Ge buffer, confirmed by (224) reflections. Ge_0.95_Si_0.05_ is 0.4% tensily strained on the Ge buffer (Fig. 2b). Note that the tensile strain in the Ge_0.95_Si_0.05_ channels can further improve the electron mobility^13,14^. The secondary ion mass spectrometry profile of the as-grown epilayers of stacked 16 Ge_0.95_Si_0.05_ channels is shown in Fig. 2c. The 16 undoped Ge_0.95_Si_0.05_ channel layers are sandwiched by 17 heavily P-doped Ge SLs. For low S/D resistance, the [P] in Ge SLs is as high as ~2 × 10^20^ cm^−3^. The minimum [P] in Ge_0.95_Si_0.05_ channels are from ~ 4 × 10^17^ cm^−3^ to ~2 × 10^19^ cm^−3^ and increases from the top to the bottom due to P diffusion during the epi growth (350 °C)^13–15^. The top channel has the lowest [P] due to the least time in CVD epitaxial growth (Fig. 2d). To mitigate this effect, the lower epi growth temperature and less time in CVD epitaxial growth are two key factors. This epilayers were grown using GeH_4_ and SiH_4_ precursors. Using high order precursors like Ge_2_H_6_ and Si_2_H_6_, the epilayers can be grown at lower temperature and enhanced growth rate can reduce the time in CVD epitaxial growth.

### Device fabrication

**Fig. 3:**
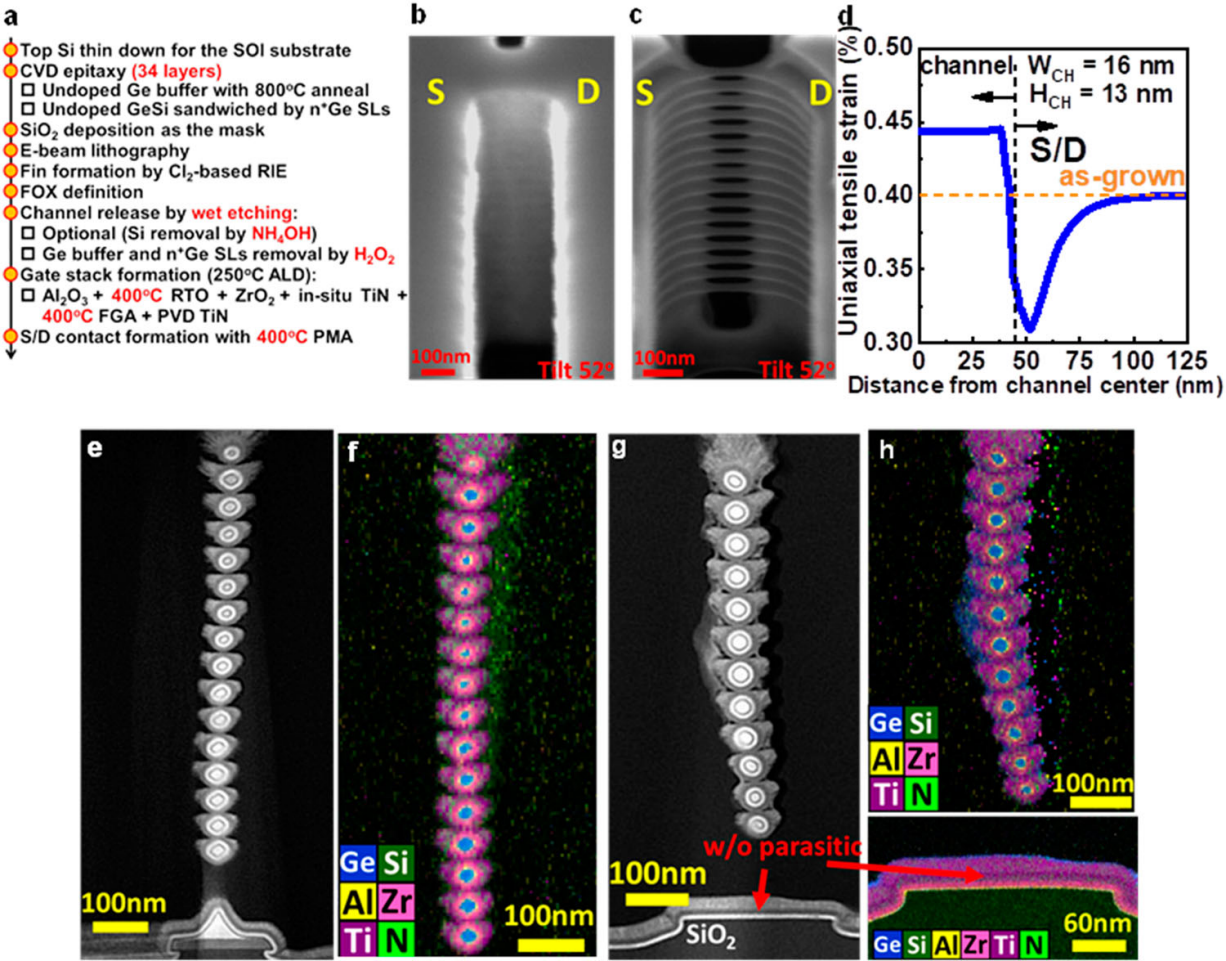
Devices fabrication of stacked 16 Ge_0.95_Si_0.05_ nanowires and stacked 12 Ge_0.95_Si_0.05_ nanowires without parasitic channel. Process flow (**a**) of the highly stacked GeSi nGAAFETs with the highlighted features (red). Tilt 52^o^ SEM after fin formation (**b**) by the Cl_2_-based RIE and channel release (**c**) by H_2_O_2_ wet etching. **d** Simulated strain of 0.44% in the GeSi channel with the average W_CH_ and H_CH_ in the microbridge structure by ANSYS. Note that the orange dash line corresponds the as-grown condition. STEM-HAADF (**e**) and EDS mapping (**f**) of the stacked 16 Ge_0.95_Si_0.05_ nanowires. STEM-HAADF (**g**) and EDS mapping (**h**) of the stacked 12 Ge_0.95_Si_0.05_ nanowires w/o parasitic channels. Note that the red arrow points parasitic channel removal. The EDS mapping shows the nanowires surrounded by the in-situ TiN to ensure the GAA structure.

The device fabrication flow of the highly stacked Ge_0.95_Si_0.05_ nanowires is summarized in Fig. 3a with the highlighted features. In this work, the S/D and channels are fabricated by the same epilayers without S/D regrowth. The doping of S/D is obtained by the n^+^Ge SLs, which are annealed during the device fabrication to have P diffusion. The growth temperature of CVD Ge_0.95_Si_0.05_ and n^+^Ge SL is maintained at 350 °C for the metastable state. The total thickness of channel layers should be less than critical thickness to avoid dislocation generation. The additional 800 °C anneal after Ge buffer growth before channel and SL epi was used to confine the misfit dislocations at Ge/SOI interface for epi quality improvement. The thick top n^+^Ge SL is designed to protect the disappearance of a top channel from etching away after channel release. After CVD epitaxy (34 layers) and SiO_2_ mask deposition by the plasma enhanced chemical vapor deposition, the e-beam lithography and Cl_2_-based reactive ion etching (RIE) were used to form the fin structures (Fig. 3b). After fin formation, the plasma enhanced chemical vapor deposition field oxide was deposited. The field oxide was patterned prior to the channel release process to prevent the oxidation and distortion of the released channels. The channel release and Ge buffer were performed by H_2_O_2_ wet etching (Fig. 3c) and parasitic SOI channels were removed by NH_4_OH + H_2_O_2_ wet etching. Note that H_2_O_2_ wet etching at room temperature was used to etch the Ge buffer and Ge SLs between the channel regions, while NH_4_OH wet etching at 75 °C^16^ was used to completely remove the SOI underneath the Ge buffer. The etching selectivity of Ge over Ge_0.95_Si_0.05_ is attributed to the heavily doped phosphorus in Si^13,14^. To investigate the strain after channel release, the strain at the center of GeSi channel is simulated by ANSYS using the average channel width (W_CH_) and channel height (H_CH_) in the microbridge structure. The uniaxial tensile strain at the center of the Ge_0.95_Si_0.05_ channel increases to 0.44% from the epitaxial strain of 0.4% to further enhance the electron mobility (Fig. 3d). The native oxide was removed by dipping with diluted HCl solution before the gate stack formation to ensure low surface roughness of Ge_0.95_Si_0.05_ channels^17^. After the 10 cycles TMA passivation^18^, the Al_2_O_3_ was conformally deposited around the nanowires by the plasma enhanced atomic layer deposition, followed by the rapid thermal oxidation at 400 °C for 1 min. ZrO_2_ and in-situ TiN by the plasma enhanced atomic layer deposition were then conformally deposited on the Al_2_O_3_. The following 400 °C forming gas annealing was used to crystallize ZrO_2_ for a large κ value. A thick TiN was then deposited as the gate metal pad by sputtering. The gate metal pad region was defined by RIE and buffered oxide etching. The thick TiN gate metal was used to protect the gate stack and to avoid top nanowires etched away during RIE. Note that RIE with CF_4_ gas is used to etch TiN/ZrO_2_/Al_2_O_3_ stacks. The S/D pad were then formed by the lithography and wet etching. The wet etching in HF solution to etch field oxide on S/D. After Pt deposited by sputtering, a lift-off process was used to pattern Pt. The 400 °C post metallization annealing were used to form the S/D contacts on Ge:P with [P] ~ 2 × 10^20^ cm^−3^ for low S/D resistance. The STEM-HAADF image (Fig. 3e) shows that the stacked 16 Ge_0.95_Si_0.05_ nanowires have the largest W_CH_ of 20 nm with all the n^+^Ge SLs are removed, indicating that the sufficient selectivity of n^+^Ge SLs over undoped Ge_0.95_Si_0.05_ channels by H_2_O_2_ wet etching. The nanowires are surrounded by the gate dielectrics and in-situ TiN to ensure the GAA structure (Fig. 3f). The STEM-HAADF image shows the stacked 12 Ge_0.95_Si_0.05_ nanowires with total removal of the SOI, Ge buffer, and n^+^Ge SLs (Fig. 3g). The EDS mapping ensures the GAA structure (Fig. 3h). Note that no S/D regrowth in our process and sacrificial layers are the doping source in S/D. The bending Fig. 3g, h is the artifact of TEM sample preparation due to floating channels affected by ion milling.

### Device performance

The highest stacked 16 Ge_0.95_Si_0.05_ nanowires have revolutionary progress, as compared with our previous works^13,14,19^. The Ge content of 95% in GeSi channels is larger than 85% to ensure the electrons populated in the high mobility L_4_ valleys^13^. In previous work^20,21^, the nanosheets have non-uniform electron distribution across the cross sections, where electron wavefunction is dense at both ends. This causes the degradation of I_ON_ per footprint as compared with the nanowires. Increasing the number of stacked channels can further enhance the I_ON_.

**Fig. 4:**
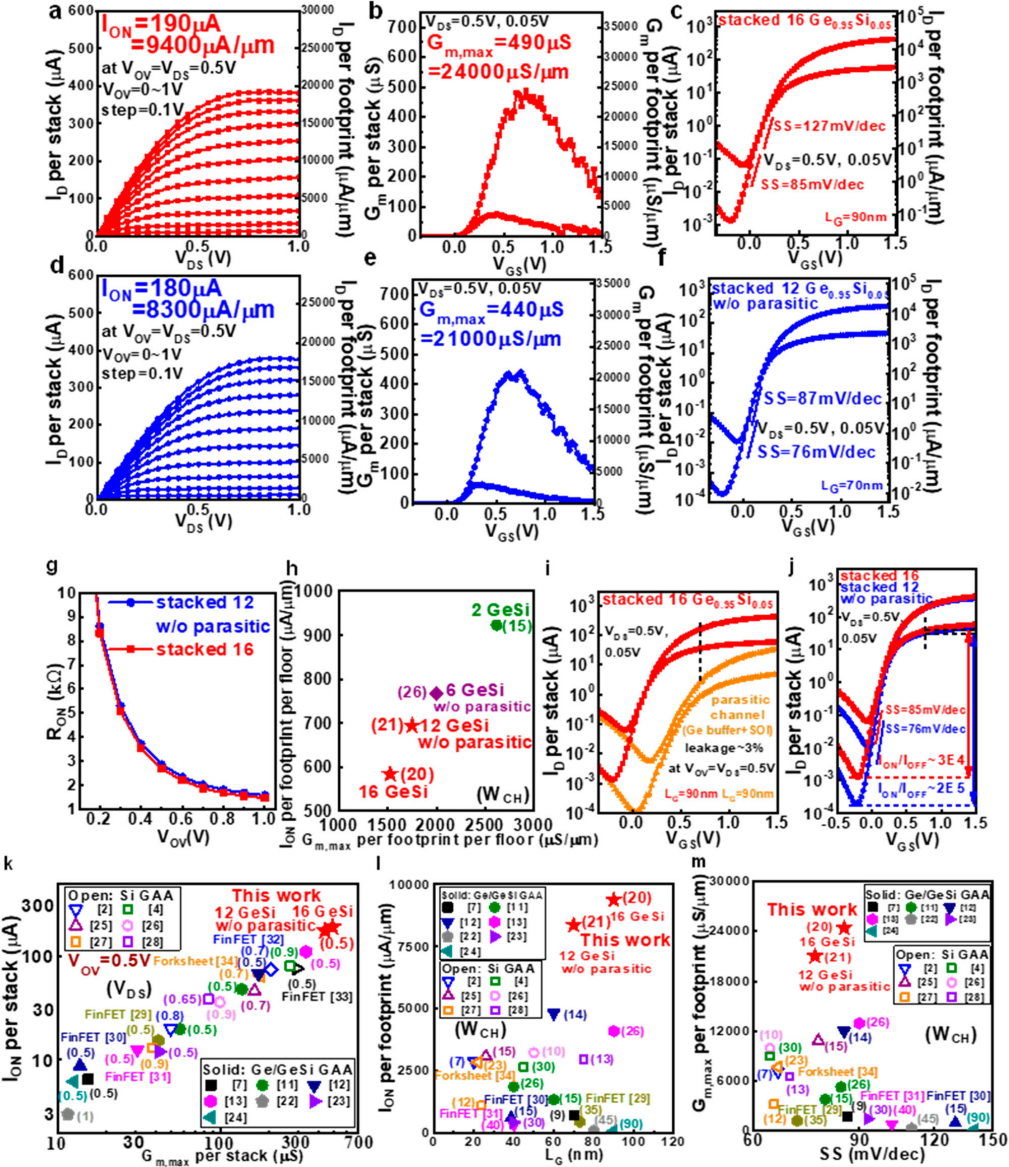
Electrical measurements and benchmarks of stacked 16 Ge_0.95_Si_0.05_ nanowires and stacked 12 Ge_0.95_Si_0.05_ nanowires without parasitic channel. I_D_-V_DS_ (**a**), G_m_-V_GS_ (**b**), and I_D_-V_GS_ (**c**) of the stacked 16 Ge_0.95_Si_0.05_ nanowires (red line). I_D_-V_DS_ (**d**), G_m_-V_GS_ (**e**), and I_D_-V_GS_ (**f**) of the stacked 12 Ge_0.95_Si_0.05_ nanowires w/o parasitic channels (blue line). **g** R_on_ comparison between stacked 16 nanowires (red line) and stacked 12 nanowires without parasitic channels (blue line). **h** Benchmarks of I_ON_ per stack per footprint vs G_m,max_ per footprint per floor. **i** I_D_-V_GS_ of stacked 16 Ge_0.95_Si_0.05_ nanowires (red line) and parasitic Ge channel (orange line). **j** I_D_-V_GS_ of stacked 16 Ge_0.95_Si_0.05_ nanowires (red line) and stacked 12 nanowires without parasitic channels (blue line). Benchmarks of I_ON_ per stack vs G_m,max_ per stack (**k**), benchmarks of I_ON_ per footprint vs L_G_ (**l**), and G_m,max_ per footprint vs SS (**m**) for Si/Ge/GeSi 3D nFETs. The stacked 16 Ge_0.95_Si_0.05_ nanowires have the high I_ON_ and G_m,max_ among reported Si/Ge/GeSi 3D nFETs. Note that solid and open symbols correspond to Ge/GeSi and Si 3D nFETs, respectively. The number in the parentheses in (**k**), and (**l**, **m**) are V_DS_ and W_CH_, respectively. The red stars correspond to this work.

The stacked 16 Ge_0.95_Si_0.05_ nanowires with L_G_ = 90 nm have the high I_ON_ of 190 μA per stack (9400 μA/μm per channel footprint) at V_OV_ = V_DS_ = 0.5 V and the high G_m,max_ of 490 μS per stack (24,000 μS/μm) at V_DS_ = 0.5 V with the SS of 85 mV/dec (Fig. 4a–c). Note that the I_ON_ and G_m,max_ per channel footprint in this work are normalized by the largest W_CH_ among the stacked channels. The removal of the parasitic channels were made possible by NH_4_OH + H_2_O_2_ etching, and the stacked 12 Ge_0.95_Si_0.05_ nanowires were still remained. The high I_ON_ of 180 μA per stack (8300 μA/μm) at V_OV_ = V_DS_ = 0.5 V and the high G_m,max_ of 440 μS per stack (21,000 μS/μm) at V_DS_ = 0.5 V with the good with the good SS of 76 mV/dec are achieved with L_G_ of 70 nm (Fig. 4d–f). The on resistance (R_ON_ ≡ V_D_/I_D_) is extracted at V_DS_ = 0.5 V and the R_ON_ vs V_OV_ is plotted in Fig. 4g. The 0.94X of R_ON_ reduction is obtained by stacked 16 nanowires as compared to stacked 12 nanowires at V_OV_ = V_DS_ = 0.5 V. The ideal R_ON_ reduction should be 12/16 = 0.75 and the difference is due to parasitic S/D resistance. Moreover, the I_OFF_ of stacked 16 nanowires is dominated by the parasitic channels (Fig. 4i). The parasitic channels have to be removed for further improvement. After the removal of all the stacked nanowires, the low leakage current (~3%) induced by the parasitic channels was measured at V_OV_ = V_DS_ = 0.5 V (Fig. 4i). The stacked 12 Ge_0.95_Si_0.05_ nanowires without parasitic channels show lower SS and larger I_ON_/I_OFF_ as compared with the stacked 16 Ge_0.95_Si_0.05_ nanowires with parasitic Ge channels by Ge buffer. The SS of stacked 12 Ge_0.95_Si_0.05_ nanowires without parasitic channels are 76 mV/dec and 87 mV/dec measured at the V_DS_ of 0.05 and 0.5 V, respectively. The SS of stacked 16 Ge_0.95_Si_0.05_ nanowires are 85 mV/dec and 127 mV/dec at V_DS_ of 0.05 and 0.5 V, respectively. The SS is reduced to 76 mV/dec from 85 mV/dec at V_DS_ = 0.05, and the I_ON_/I_OFF_ is improved to ~2 × 10^5^ from ~3 × 10^4^ after removing parasitic channels (Fig. 4j).

### Benchmarks

The stacked 16 Ge_0.95_Si_0.05_ FETs reach the high I_ON_ per stack of 190 μA at V_OV_ = 0.5 V and the high G_m,max_ per stack of 490 μS among reported Si/Ge/GeSi 3D nFETs (Fig. 4k)^2,4,7,11–13,22–34^. Note that the V_DS_ to benchmark I_ON_ and G_m,max_ is indicated in the parentheses. Ideally, I_ON_ and G_m_ should be enhanced to be 16/12 = 4/3 as the floor number increase from 12 to 16 if S/D resistance is negligible. However, the S/D has neither a sufficient doping concentration nor a sufficient area for metal contact, and the parasitic S/D resistance leads to decreasing I_ON_ and G_m_ per floor with increasing floor number (Fig. 4h). However, the I_ON_ and G_m_ still increases with increasing floor number (Fig. 4k). The benchmarks of I_ON_ per footprint vs L_G_ and G_m,max_ per footprint vs SS are shown in Fig. 4l, m^2,4,7,11–13,22–34^, respectively. The high I_ON_ per footprint of 9400 μA/μm at V_OV_ = 0.5 V and the high G_m,max_ per footprint of 24,000 μS/μm are achieved among reported Si/Ge/GeSi 3D nFETs. Note that the channel width (W_CH_) is indicated in the parentheses in Fig. 4l, m.

### Improved current distribution, capacitance, and delay by the TCAD simulation

**Fig. 5:**
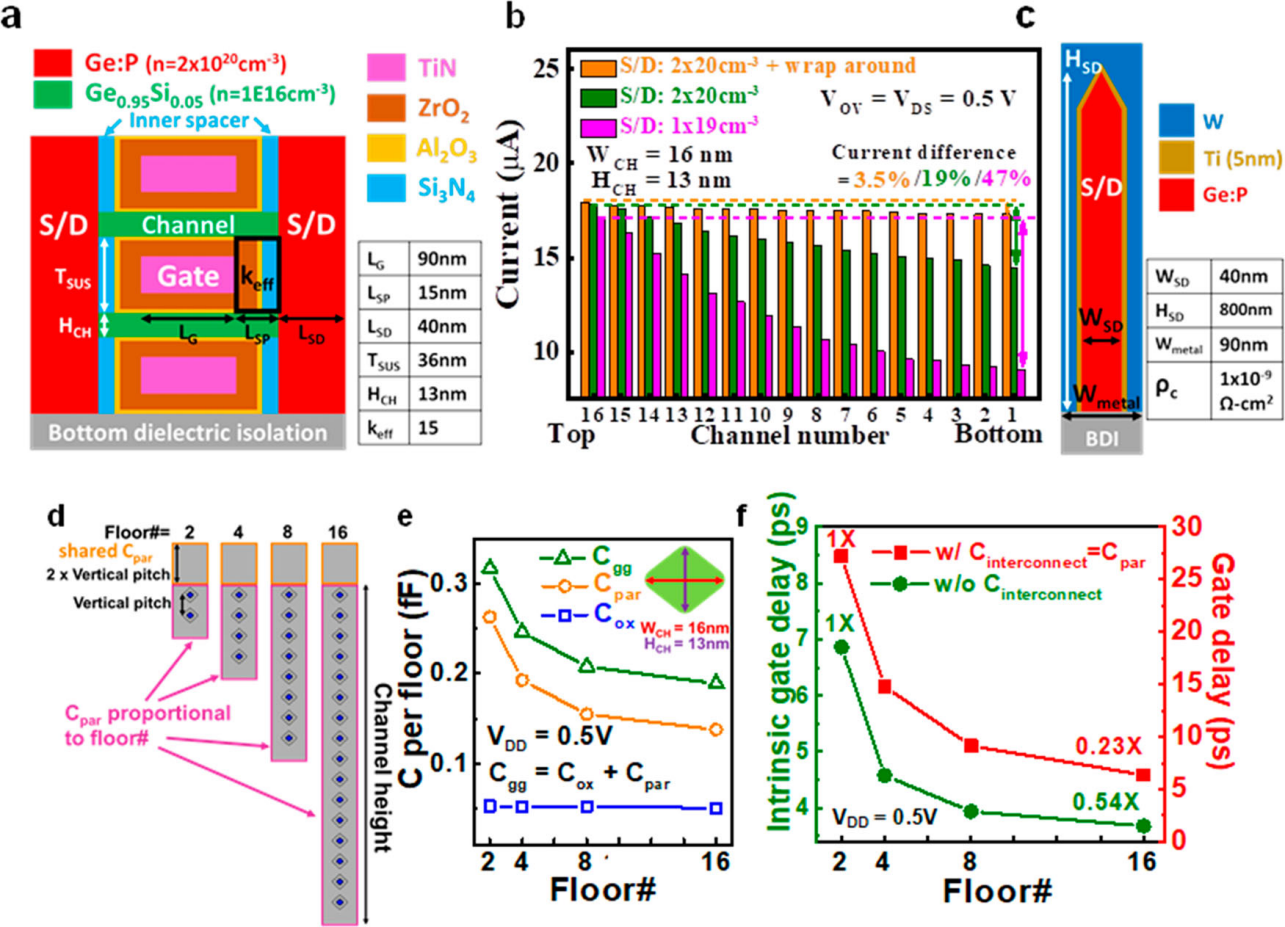
Simulation of stacked 16 Ge_0.95_Si_0.05_ nanowire FETs. **a** Schematic of simulated device structure. **b** Simulated current vs channel number. The S/D doping of 1.3 × 10^19^ cm^−3^ (magenta bars), 2 × 10^20^ cm^−3^ (olive bars), and 2 × 10^20^ cm^−3^ with warp around contacts (orange bars) are used. **c** Schematic of wrap around contact structure at S/D region. **d** Total parasitic capacitance (C_par_) consisting of shared C_par_ (orange) and C_par_ proportional to floor# (purple) with the floor# of 2/4/8/16. **e** Total gate capacitance (C_gg_, olive line), C_par_ (orange line), intrinsic gate capacitance (C_ox_, blue line) per floor and **f** intrinsic gate delay (olive)/gate delay (red) improvement vs floor# using the average W_CH_ = 16 nm and H_CH_ = 13 nm. The current difference between top channel and bottom channel is only 3.5% for the S/D doping of 2 × 10^20^ cm^−3^ with wrap around contacts. The effective dielectric constant (κ_eff_ = 15) the average of 5 nm Si_3_N_4_ inner spacer (κ = 7.2), 1 nm Al_2_O_3_ (κ = 9), and 9 nm ZrO_2_ (κ = 46).

The industrial device structure (Fig. 5a) is used for the simulation of current, capacitances, and delay by the TCAD^35^. The simulated current vs channel number of the stacked 16 Ge_0.95_Si_0.05_ FETs is shown in Fig. 5b. Note that all the current is normalized with respect to the current of the top channel (channel number 16). The series resistance impacts the transistor performance. Three types of S/D are considered in the simulation including S/D doping of 1.3 × 10^19^ cm^−3^, S/D doping of 2 × 10^20^ cm^−3^, and wrap around contact with S/D doping of 2 × 10^20^ cm^−3^ (Fig. 5b). For the S/D doping of 1.3 × 10^19^ cm^−3^, the current reduction from the top channel to the bottom channel is as high as 47%. However, for S/D doping of 2 × 10^20^ cm^−3^ and the wrap around contact, the series resistance effect can be reduced, leading to only a 3.5% current reduction from the top channel to the bottom channel. Thus, the total current can be proportional to floor#.

The total gate capacitance (C_gg_) is the sum of intrinsic gate capacitance (C_ox_) and parasitic capacitance (C_par_), i.e., C_gg_ = C_ox_+C_par_. In our simulation structure, the effective dielectric constant (κ_eff_) = 15 is used in the inner spacer considering 5 nm Si_3_N_4_ (κ = 7.2), 1 nm Al_2_O_3_ (κ = 9), and 9 nm ZrO_2_ (κ = 46) (Fig. 5a). Note that 1 nm Al_2_O_3_ and 9 nm ZrO_2_ used in the inner spacer are due to the conformal deposition of ALD oxide. The overlap area between the gate metal and S/D metal is the main contribution to C_par_. For the overlap area between the gate and S/D, the gate metal width and S/D width are 90 nm (Fig. 5c), while the gate metal height is the sum of channel height (proportional to floor number) and additional 2 vertical pitches above the channel height (Fig. 5d). The gate metal height is 40 nm lower than the S/D metal (Fig. 5a), which can reduce the gate to S/D overlap area, similar to our previous work^36^. The gate to S/D overlap area above the top channel (2 vertical pitches) is shared by total floors. Therefore, the C_par_ per floor decreases as the floor number increases (Fig. 5e), and total C_par_ still increases with increasing floor#. Besides, the intrinsic gate capacitance (C_ox_) per floor remains similar as floor# increases from 2 to 16, and is smaller than C_par_ per floor. Thus, C_gg_ per floor decreases as the floor# increases, similar to the trend of C_par_ per floor (Fig. 5e).

Due to the small 3.5% current decrease (Fig. 5b) and the large decrease (40%) of C_gg_ per floor (Fig. 5e), the intrinsic gate delay (Eq. (1))^37^ of the floor#=16 is 0.54X of the floor#=2 (Fig. 5f). Moreover, considering the interconnect capacitance (C_interconnect_=C_par_), the gate delay (Eq. (2))^38^ of the floor#=16 is improved to be 0.23X as compared to floor#=2.

1 $$\,{Intrinsic}\,{gate}\,{delay}=\frac{{{V}_{{DD}}C}_{{gg}}}{2{I}_{{eff}}}=\frac{ {V}_{{DD}} (C_{{ox}}+{C}_{par})} {2{I}_{eff}}$$

2 $${Gate}\,{delay}=\frac{{V}_{{DD}}(C_{{gg}}+{C}_{{interconnect}})}{2{I}_{eff}}$$

## Conclusions

The isotropic wet etching with sufficient selectivity and sophisticated 34 epilayers were used to fabricate the stacked 16 Ge_0.95_Si_0.05_ nanowire FETs. The highly stacked nanowire FETs are used to enhance drive current and transistor density due to its small footprint. The wrap around contacts are useful to reduce the current difference between the channels. With the proper design of transistor height, the gate delay can be also improved.

## Methods

### Device structure and epilayer design

The stacked 16 channels without S/D regrowth is demonstrated. The doping of S/D is obtained by the heavily P-doped Ge sacrificial layers, which are annealed during the device fabrication to have P diffusion. The epilayers were grown in a rapid thermal chemical vapor deposition system with a cold-wall quartz chamber using modified ASM Epsilon 2000 PLUS. The precursor of Ge_0.95_Si_0.05_ channels and P-doped Ge SLs are SiH_4_, GeH_4_, and PH_3_.

### Material analysis of epilayers

The as-grown epilayers with stacked 16 Ge_0.95_Si_0.05_ channels were analyzed by the high-resolution X-ray diffraction with ω − 2θ scan of (004) reflections. The reciprocal space mapping was used to further analyze the strain of epitaxial layers. The doping profile of the as-grown epilayers of stacked 16 Ge_0.95_Si_0.05_ channels was analyzed by secondary ion mass spectrometry.

### Device fabrication

After epitaxy, the hard mask was deposited to protect epilayers by the plasma enhanced chemical vapor deposition using Oxford 100 PECVD cassette system. The gate lengths of stacked 16 Ge_0.95_Si_0.05_ nanowire FETs and stacked 12 Ge_0.95_Si_0.05_ nanowire FETs without parasitic channels are 90 nm and 70 nm defined by the E-beam lithography using VISTEC SB3050-2. After E-beam lithography, the SiO_2_ hard mask and fin formation are formed by the CHF_3_-based and Cl_2_-based RIE using LAM 2300 Etcehr Exelan Flex, respectively. The channel release was performed by H_2_O_2_ wet etching and parasitic channels (Ge buffer + SOI) were removed by NH_4_OH + H_2_O_2_ wet etching. Note that NH_4_OH wet etching at 75 °C^16^ was used to completely remove the SOI underneath the Ge buffer while H_2_O_2_ wet etching was used to etch the Ge buffer and Ge SLs between the channel regions. After channel release, the stacked channels were checked by the SEM using FEI Nova 600 Nanolab Dual-Beam FIB. The gate stack of devices used in this work consisted of layers of Al_2_O_3_, ZrO_2_, and TiN by the plasma enhanced atomic layer deposition using Cambridge NanoTech Fiji ALD system. Note that the precursor of Al_2_O_3_, ZrO_2_, and TiN are trimethylaluminum (TMA), Tetrakis(dimethylamino)zirconium (TDMAZr), and Tetrakis(dimethylamino)titanium (TDMAT). The RIE using Samco RIE-10NR. The S/D contact was patterned and etched in HF solution, and was sputtered Pt.

### Electrical characterization

I_D_-V_GS_ and I_D_-V_DS_ were performed on the stacked 16 Ge_0.95_Si_0.05_ nanowire FETs and stacked 12 Ge_0.95_Si_0.05_ nanowire FETs without parasitic channel with a Keithley 4200-SCS Semiconductor Analyzer.

### TCAD simulation

Current, capacitance, and delay were simulated considering Masetti mobility model^39^. Capacitance is extracted by the small-signal AC simulation. C_par_ is extracted at off-state. C_gg_ is extracted at V_ov_ = V_DS_ = 0.5 V. Note that C_ox_=C_gg_ - C_par_^36,40^.

## Data Availability

The data that support the plots within this paper and other findings of this study are available from the corresponding author upon request.

## References

[1] Bangsaruntip, S. et al. High performance and highly uniform gate-all-around silicon nanowire MOSFETs with wire size dependent scaling. In *Proc. IEEE International Electron Devices Meeting* (*IEDM*) pp. 297 (IEEE, 2009).

[2] Lauer, I. et al. Si nanowire CMOS fabricated with minimal deviation from RMG FinFET technology showing record performance. In *Symposium on VLSI Technology Digest of Technical Papers* pp. 140 (IEEE, 2015).

[3] Loubet, N. et al. Stacked nanosheet gate-all-around transistor to enable scaling beyond FinFET. In *Symposium on VLSI Technology Digest of Technical Papers* pp. 230 (IEEE, 2017).

[4] Barraud, S. et al. 7-levels-stacked nanosheet GAA transistors for high performance computing. In *Symposium on VLSI Technology Digest of Technical Papers* pp. TC1-2 (IEEE, 2020).

[5] Liu, M. Unleashing the future of innovation. In *International Solid-State Circuits Conference (ISSCC)*, Plenary Session 1.1 (IEEE, 2021).

[6] Mii, Y.-J. Semiconductor innovations from device to system. In *Symposium on VLSI Technology Digest of Technical Papers* P2-2 (IEEE, 2022).

[7] van Dal, M. J. H. et al. Ge CMOS gate stack and contact development for vertically stacked lateral nanowire FETs. In *Proc. IEEE International Electron Devices Meeting* (*IEDM*) pp. 492–495 (IEEE, 2018).

[8] Tsai, C.-E. et al. Highly stacked 8 Ge_0.9_Sn_0.1_ nanosheet pFETs with ultrathin bodies (~3 nm) and thick bodies (~30 nm) featuring the respective record I_ON_/I_OFF_ of 1.4 × 10^7^ and record I_ON_ of 92 μA at V_OV_ = V_DS_ = -0.5 V by CVD epitaxy and dry etching. In *Proc. IEEE International Electron Devices Meeting* (*IEDM*) pp. 569–572 (IEEE, 2021).

[9] Huang B-W (2022). Highly stacked GeSn nanosheets by CVD epitaxy and highly selective isotropic dry etching. IEEE Trans. Electron Devices.

[10] Tsai, C.-E. et al. Nearly ideal subthreshold swing and delay reduction of stacked nanosheets using ultrathin bodies. In *Symposium on VLSI Technology Digest of Technical Papers* pp. 401–402 (IEEE, 2022).

[11] Tu, C.-T. et al. First vertically stacked tensily strained Ge_0.98_Si_0.02_ nGAAFETs with no parasitic channel and L_G_ = 40 nm featuring record I_ON_ = 48 µA at V_OV_ = V_DS_ = 0.5 V and record G_m,max_(µS/µm)/SS_SAT_(mV/dec) = 8.3 at V_DS_ = 0.5 V. In *Proc. IEEE International Electron Devices Meeting* (*IEDM*) pp. 681–684 (IEEE, 2019).

[12] Chen Y-R (2022). I_ON_ enhancement of Ge_0.98_Si_0.02_ nanowire nFETs by high-κ dielectrics. IEEE Electron Device Lett..

[13] Liu, Y.-C. et al. First highly stacked Ge_0.95_Si_0.05_ nGAAFETs with record I_ON_ = 110 μA (4100 μA/μm) at V_OV_ = V_DS_ = 0.5 V and high G_m,max_ = 340 μS (13000 μS/μm) at V_DS_ = 0.5 V by wet etching. In *Symposium on VLSI Technology Digest of Technical Papers* pp. T15-2 1-2 (IEEE, 2021).

[14] Liu Y-C (2021). Highly stacked GeSi nanosheets and nanowires by low-temperature epitaxy and wet etching. IEEE Trans. Electron Devices.

[15] Hsieh, W.-H. et al. Diffusion and segregation in highly stacked Ge_0.9_Sn_0.1_/Ge:B and Ge_0.95_Si_0.05_/Ge:P epilayers. *241st ECS Meeting Abstracts MA2022-01* 1284 (2022).

[16] Wang F (1997). Highly selective chemical etching of Si vs. Si_1-x_Ge_x_ using NH_4_OH solution. J. Electrochem. Soc..

[17] Sun S (2006). Surface termination and roughness of Ge(100) cleaned by HF and HCl solutions. Appl. Phys. Lett..

[18] Lee, T.-E. et al. Improvement of SiGe MOS interface properties with a wide range of Ge contents by using TiN/Y_2_O_3_ gate stacks with TMA passivation. In *Symposium on VLSI Technology Digest of Technical Papers* pp. T9-5 100–101 (IEEE, 2019).

[19] Thomas S (2021). Germanium nanowire transistors stack up. Nat. Electron..

[20] Huang, Y.-S. et al. First demonstration of uniform 4-stacked Ge_0.9_Sn_0.1_ nanosheets with record I_ON_ =73µA at V_OV_=V_DS_= −0.5V and low noise using double Ge_0.95_Sn_0.05_ caps, dry etch, low channel doping, and high S/D doping. In *Proc. IEEE International Electron Devices Meeting* (*IEDM*) pp. 2.4.1–2.4.4 (IEEE, 2020).

[21] Tu C-T (2021). Uniform 4-Stacked Ge_0.9_Sn_0.1_ nanosheets using double Ge_0.95_Sn_0.05_ caps by highly selective isotropic dry etch. IEEE Trans. Electron Devices.

[22] Lee, Y.-J. et al. Diamond-shaped Ge and Ge_0.9_Si_0.1_ gate-all-around nanowire FETs with four {111} facets by dry etch technology. In *Proc. IEEE International Electron Devices Meeting* (*IEDM*) pp. 382–385 (IEEE, 2015).

[23] Wu, H. et al. First demonstration of Ge nanowire CMOS circuits: lowest SS of 64 mV/dec, highest g_max_ of 1057 μS/μm in Ge nFETs and highest maximum voltage gain of 54 V/V in Ge CMOS inverters. In *Proc. IEEE International Electron Devices Meeting* (*IEDM*) pp. 2.1.1–2.1.4 (IEEE, 2015).

[24] Chu C-L (2018). Stacked Ge-nanosheet GAAFETs fabricated by Ge/Si multilayer epitaxy. IEEE Electron Device Lett..

[25] Ritzenthaler, R. et al. Vertically stacked gate-all-around Si nanowire CMOS transistors with reduced vertical nanowires separation, new work function metal gate solutions, and DC/AC performance optimization. In *Proc. IEEE International Electron Devices Meeting* (*IEDM*) pp. 508–511 (IEEE, 2018).

[26] Barraud, S. et al. Tunability of parasitic channel in gate-all-round stacked nanosheets. In *Proc. IEEE International Electron Devices Meeting* (*IEDM*) pp. 500–503 (IEEE, 2018).

[27] Mertens, H. et al. Vertically stacked gate-all-around Si nanowire transistors: key process optimizations and ring oscillator demonstration. In *Proc. IEEE International Electron Devices Meeting* (*IEDM*) pp. 828–831 (IEEE, 2017).

[28] Huang, C.-Y. et al. 3-D Self-aligned stacked NMOS-on-PMOS nanoribbon transistors for continued Moore’s Law scaling. In *Proc. IEEE International Electron Devices Meeting* (*IEDM*) pp. 425–428 (IEEE, 2020).

[29] Arimura, H. et al. A record Gm_SAT_/SS_SAT_ and PBTI reliability in Si-passivated Ge nFinFETs by improved gate stack surface preparation. In *Symposium on VLSI Technology Digest of Technical Papers* pp. T9-1 92–93 (IEEE, 2019).

[30] Mitard, J. et al. First demonstration of 15nm-WFIN inversion-mode relaxed-germanium n-FinFETs with Si-cap free RMG and NiSiGe source/drain. In *Proc. IEEE International Electron Devices Meeting* (*IEDM*) pp. 418–421 (IEEE, 2014).

[31] van Dal, M. J. H. et al. Ge n-channel FinFET with optimized gate stack and contacts. In *Proc. IEEE International Electron Devices Meeting* (*IEDM*) pp. 235–238 (IEEE, 2014).

[32] Wu, S.-Y. et al. Demonstration of a sub-0.03 µm^2^ High Density 6-T SRAM with scaled bulk FinFETs for mobile SOC applications beyond 10nm node. In *Symposium on VLSI Technology Digest of Technical Papers* pp. T9.1 92–93 (IEEE, 2016).

[33] Rachmady, W. et al. 300mm heterogeneous 3D integration of record performance layer transfer germanium PMOS with silicon NMOS for low power high performance logic applications. In *Proc. IEEE International Electron Devices Meeting* (*IEDM*) pp. 697–700 (IEEE, 2019)

[34] Mertens, H. et al. Forksheet FETs for advanced CMOS scaling: forksheet-nanosheet co-integration and dual work function metal gates at 17nm N-P Space. In *Symposium on VLSI Technology Digest of Technical Papers* pp. T2-1 1–2 (IEEE, 2021).

[35] TCAD Sentaurus Suite, version T-2022.03, Synopsys Inc., Mountain View, CA (2022).

[36] Lin H-C (2021). RF performance of stacked Si nanosheet nFETs. IEEE Trans. Electron Devices.

[37] Yoon J (2019). Bottom oxide bulk FinFETs without punch-through-stopper for extending toward 5-nm node. IEEE Access.

[38] Huynh-Bao, T. et al. Statistical timing analysis considering device and interconnect variability for BEOL requirements in the 5-nm node and beyond. *IEEE Transaction on Very Large Scale Integration (VLSI) Systems* **25** (2017).

[39] Masetti G (1983). Modeling of carrier mobility against carrier concentration in arsenic-, phosphorus-, and boron-doped silicon. IEEE Trans. Electron Devices.

[40] Lin H-C (2022). RF performance of stacked Si nanosheets/nanowires. IEEE Electron Device Lett..

